# The two complete plastomes from *Scrophularia* (Scrophulariaceae): *Scrophularia buergeriana* and *S. takesimensis*

**DOI:** 10.1080/23802359.2016.1225528

**Published:** 2016-09-18

**Authors:** Dong-Keun Yi, Ki-Joong Kim

**Affiliations:** School of Life Sciences, Korea University, Seoul, Korea

**Keywords:** Scrophulariaceae, *Scrophularia buergeriana*, *Scrophularia takesimensis*, plastome

## Abstract

The plastome sequences of *Scrophularia buergeriana* and *S. takesimensis* are completed in family Scrophulariaceae. The structure of two *Scrophularia* plastomes shows similar characteristic with the typical plastome of angiosperm. The lengths of two plastomes are 153,631bp and 152,436bp, respectively. They are divided into LSC region (84,454bp and 83,542bp) and SSC region (17,929bp and 17,938bp) by two IR regions (25,624bp and 25,478bp). Both plastomes contain 113 genes including 79 protein coding genes, 30 tRNA genes and 4 rRNA genes. Eight protein-coding, seven tRNA and four rRNA genes are duplicated in the IR regions. Eighteen genes have one or two intron(s). The overall A-T contents of two genomes are 62.0% and 61.9%, respectively. The A-T content in the non-coding (both 64.5%) is higher than in the coding (60.2% and 60.1%) region. Forty-four and forty-one simple sequence repeat (SSR) loci are identified in the *S. buergeriana* and *S. takesimensis* plastomes, respectively. In phylogenetic analysis, the genus *Scrophularia* shows closed relationship with Plantaginaceae.

Genus *Scrophularia* in family Scrophulariaceae which is commonly called as ‘figwort’ includes about 200 species (Carlbom 1969; Scheunert & Heubl 2011). Most *Scrophularia* species are native in Asia and several species are used in traditional oriental medicine. We report the plastomes of *Scrophularia buergeriana* Miquel and *S. takesimensis* Nakai. *Scrophularia buergeriana* is occasionally cultivated in Korea for oriental medicine and *S. takesimensis* is Korean endemic species (Nakai 1949). The plant material of *S. buergeriana* was collected from a single individual, which was cultivated at the Agriculture Technology Center of Andong city in Korea and *S. takesimensis* was collected in the natural habitat at Ulleung island of Korea, respectively. The voucher specimens are deposited in the Korea University Herbarium (KUS2014-0384, KUS2014-1538). Total DNAs are isolated by standard CTAB extraction method (Doyle & Doyle 1990) and deposited in the Plant DNA Bank of Korea (PDBK 2014-0384, PDBK 2014-1538). Plastome sequences were analyzed using Illumina MiSeq (Illumina, San Diego, CA), and assembled by Geneious 8.1.7 (http://www.geneious.com, Kearse et al. 2012). The complete plastome sequence is available from NCBI database under the accession numbers of KP718626 and KP718628, respectively.

The length of complete plastomes of *S. buergeriana* and *S. takesimensis* are 153,631bp and 152,436bp, respectively. The plastome of *S. buergeriana* is composed of 84,454bp of LSC region, 17,929bp of SSC region, and 25,624bp of two IR regions, whereas the genome of *S. takesimensis* is composed of 83,542bp of LSC region, 17,938bp of SSC region and 25,478bp of two IR regions. The plastome sequences of *S. takesimensis* were published recently (Choi & Park 2016; NC_026202). Our *S. takesimesis* plastome (KP718628) is 11 bp longer than the published one (NC_026202). Ten indels, seven polymorphisms and one small inversion are observed between two sequences of *S. takesimensis*.

Plastomes of *S. buergeriana* and *S. takesimensis* are composed of 113 individual genes which included 79 protein-coding genes, 30 transfer RNA genes, and 4 ribosomal RNA genes. Among them, eight protein-coding genes, seven tRNA genes, and four rRNA genes are duplicated on the IR regions. Fifteen genes have one two introns. Three genes such as *rps12*, *clpP*, and *ycf3* have two introns (Shinozaki et al. 1986; Kim & Lee 2004; Yi & Kim 2012).

The major portions of the *S. buergeriana* and *S. takesimensis* plastomes are gene-coding regions (57.7% and 58.1%). The overall A-T contents of two genomes are 62.0% and 61.9%, respectively. The A-T content in the non-coding region (both 64.5%) is higher than in the coding (60.2% and 60.1%) regions. The A-T contents of the IR region (56.8%) is lower than the LSC (63.9%) and (SSC 67.8%) regions. These features are similar to that of typical angiosperms and other published Lamiales (Shinozaki et al. 1986; Kim & Lee 2004; Yi and Kim 2012; Wicke et al. 2013; Zhu et al. 2014; Welch et al. 2016;). Forty-four and 41 SSR loci that repeated more than 10 times are identified in the *S. buergeriana* and *S. takesimensis* plastomes, respectively.

For the phylogenetic analysis, we assembled the 56 complete cp DNA sequences from the Lamiales clade and two outgroups (Rubiaceae in Gentianales). A total of 79 protein CDSs including *rrn* genes were aligned for the 56 selected taxa. The aligned data matrix consists of a total of 84,978bp. An ML tree was obtained with − lnL =459,584.0962 using the GTR + G + I base substitution model (Figure 1) which is similar to the APG system, the genus *Scrophularia* of Scrophulariaceae from a basal clade of other six families of Lamiales. Plantaginaceae is sister to the family Scrophulariaceae (Olmstead et al. 2001; Bremer et al. 2002; Olmstead et al. 2009; The angiosperm phylogeny group 2016).

**Figure 1.**
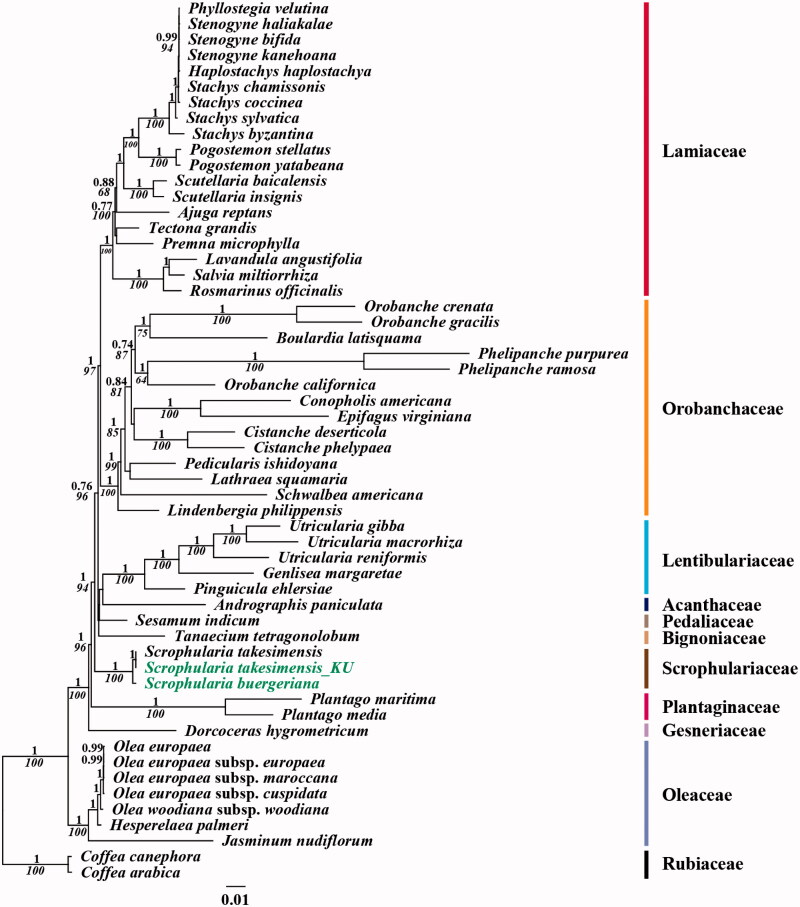
Chloroplast phylogenetic tree of Lamiales. A maximum-likelihood tree (−lnL= 459,584.0962) inferred from analysis of alignment data containing 79 coding genes in 56 chloroplast genome sequences by use of the GTR + Γ+I model. The numbers above and below each node indicate the Bayesian support percentages and bootstrap value, respectively. Genbank accession numbers for each taxa are *Ajuga reptans *(NC_023102), *Andrographis paniculata *(NC_022451), *Boulardia latisquama *(NC_025641), *Cistanche deserticola* (NC_021111), *C. phelypaea* (NC_025642), *Coffea arabica *(NC_008535), *C. canephora *(NC_030053), *Conopholis americana *(NC_023131), *Dorcoceras hygrometricum *(NC_016468), *Epifagus virginiana *(NC_001568), *Genlisea margaretae *(NC_025652), *Haplostachys haplostachya *(NC_029819), *Hesperelaea palmeri *(NC_025787), *Jasminum nudiflorum *(NC_008407), *Lathraea squamaria *(NC_027838).
